# Imp is expressed in INPs and newborn neurons where it regulates neuropil targeting in the central complex

**DOI:** 10.1186/s13064-023-00177-9

**Published:** 2023-11-29

**Authors:** Jordan A. Munroe, Chris Q. Doe

**Affiliations:** Institute of Neuroscience, Howard Hughes Medical Institute, Univ. of Oregon, Eugene, OR 97403 USA

## Abstract

The generation of neuronal diversity remains incompletely understood. In *Drosophila,* the central brain is populated by neural stem cells derived from progenitors called neuroblasts (NBs). There are two types of NBs, type 1 and 2. T1NBs have a relatively simple lineage, whereas T2NBs expand and diversify the neural population with the generation of intermediate neural progenitors (INPs), contributing many neurons to the adult central complex, a brain region essential for navigation. However, it is not fully understood how neural diversity is created in T2NB and INP lineages. Imp, an RNA-binding protein, is expressed in T2NBs in a high-to-low temporal gradient, while the RNA-binding protein Syncrip forms an opposing gradient. It remains unknown if Imp expression is carried into INPs; whether it forms a gradient similar to NBs; and whether INP expression of Imp is required for generating neuronal identity or morphology. Here, we show that Imp/Syp are both present in INPs, but not always in opposing gradients. We find that newborn INPs adopt their Imp/Syp levels from their parental T2NBs; that Imp and Syp are expressed in stage-specific high-to-low gradients in INPs. In addition, there is a late INP pulse of Imp. We find that neurons born from old INPs (E-PG and PF-R neurons) have altered morphology following both Imp knock-down and Imp overexpression. We conclude that Imp functions in INPs and newborn neurons to determine proper neuronal morphology and central complex neuropil organization.

**Supplementary Information:**

The online version contains supplementary material available at 10.1186/s13064-023-00177-9.

## Introduction

**Fig. 1:**
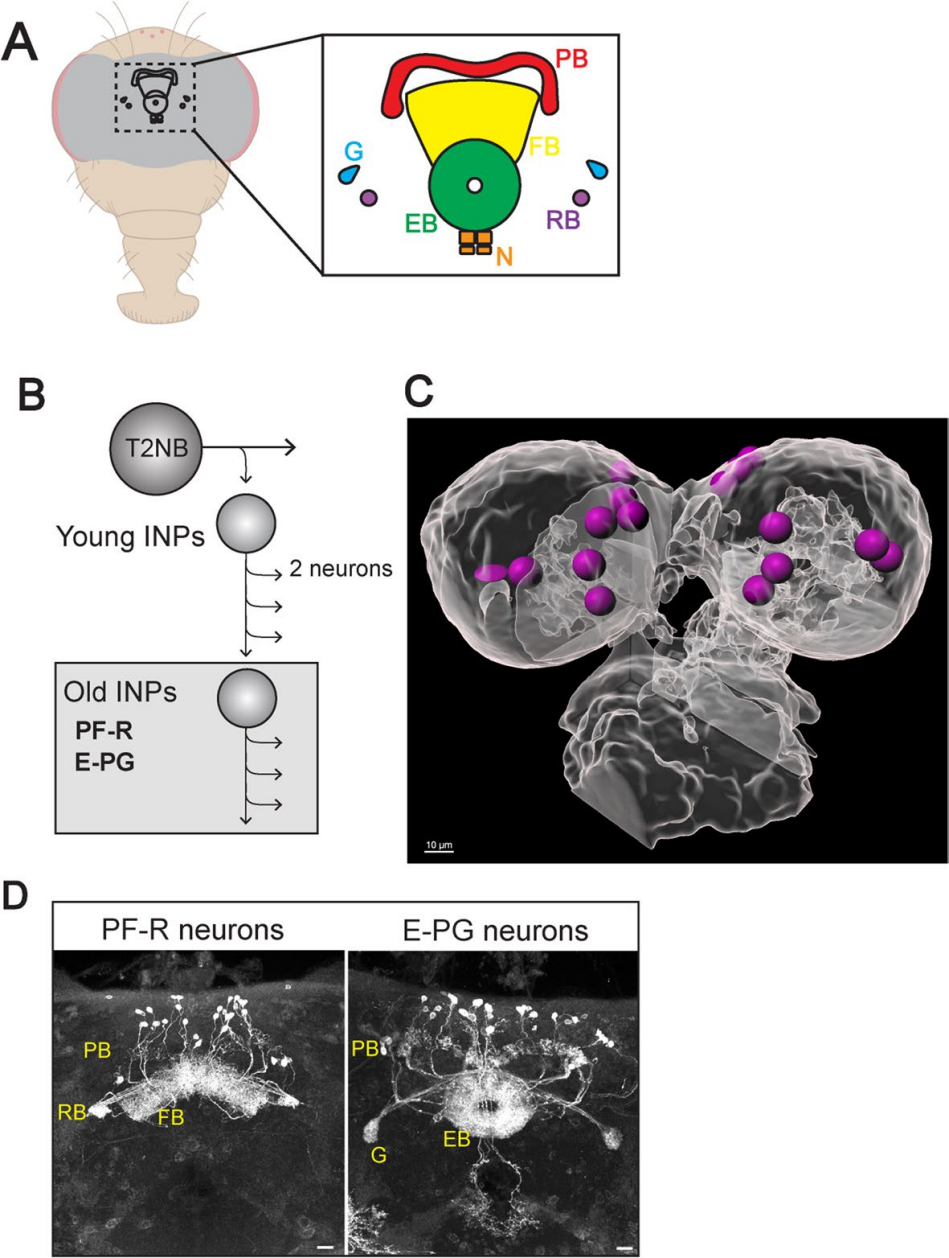
The central complex E-PG and PF-R neurons arise from T2NBs. **A** The central complex consists of six neuropils: protocerebral bridge (PB, red), fan-shaped body (FB, yellow), ellipsoid body (EB, green), noduli (N, orange), round body (RB, purple), and gall (G, blue). **B** T2NB neuroblast division pattern. E-PG and PF-R neurons arise from old INPs. **C** Still frame from Supplemental Video 1. T2NBs identified by pnt-gal4 UAS-GFP and represented as magenta spheres to show position in the 60h ALH central brain. Dorsal view, anterior up. Scale bar 10um. **D** Maximum intensity projections of confocal imaged PF-R and E-PG neurons in the adult *Drosophila* brain. Scale bar 20 μm

Across the animal kingdom a functioning brain and nervous system allows animals to perform complex behaviors. Here we use *Drosophila melanogaster* as a model to understand how the neural diversity in the brain is generated. The *Drosophila* brain develops from neural stem cells, called neuroblasts (NBs )[9]. There are two types of NBs: Type 1 NBs (T1NBs) undergo asymmetrical division to produce ganglion mother cells (GMCs) that divide to produce a pair of neurons [24]; there are about 100 type 1 NBs per larval brain lobe. Type 2 NBs (T2NBs) have a more complex lineage, undergoing a series of asymmetric divisions to produce smaller Intermediate Neural Progenitors (INPs); each INP subsequently undergoes 4–5 molecularly asymmetric divisions to produce a series of GMCs, and finally each GMC produces a pair of post-mitotic sibling neurons [3–5]; there are 16 lineages per brain (Fig. 1C, Supp. Video 1). Thus, each T1NB lineage produces ~ 100 neurons, whereas each T2NB lineage produces 500+ neurons [3–5, 13, 21, 35]. In addition, T1 and T2NBs are molecularly distinct: T1NBs are Asense (Ase) + Pointed (Pnt)-negative, whereas T2NBs are Pnt + Ase- [5, 37]. Both types of NBs are positive for the pan-NB marker Deadpan (Dpn). Interestingly, both T2NBs and outer radial glial cells (oRGs) in the primate neocortex have a cell lineage containing INPs [11].

Progeny of T2NBs are major contributors to the intrinsic neurons of the central complex (CX), an evolutionarily conserved brain region in all insects assayed to date [6], and has been proposed to be similar to the basal ganglia in humans [26]. The CX is critical for celestial navigation in both walking and flying behaviors [10, 12, 23, 30, 33]. The CX is a collection of six neuropils, or areas of dense synaptic connections. These neuropils are the protocerebral bridge (PB), fan-shaped body (FB), ellipsoid body (EB), noduli (N), gall (G), and round bodies (RB) [30] (Fig. 1). Different types of neurons connect different combinations of these neuropils. Here, we focus on two types of CX neurons: PF-Rs (25–30 neurons) and E-PGs (35–40 neurons) [27, 34].

T2NBs are formed in the embryonic brain [1, 32], undergo several divisions, and then both T2NBs and INPs undergo a period of quiescence [7, 14, 19]. They exit quiescence 12-36 h after larval hatching (ALH; subsequently all larval ages are given as ALH) [19]. As T2NBs divide and age, they express different temporal factors in a process called temporal patterning. Two of these factors are the RNA-binding proteins insulin-like growth factor-II mRNA-binding protein (Imp), and Syncrip (Syp) [20, 28]. These two RNA-binding proteins are found in opposing temporal gradients within T1 and T2NBs throughout larval stages [20, 28] (Fig. 1). Imp has high expression early in T2NBs (0-60 h), whereas Syp has an opposite expression pattern, late in T2NBs (60-120 h) [16, 20, 28]. In addition, Imp and Syp have opposing roles in regulating NB proliferation: Imp promotes NB proliferation by stabilizing Myc and Chinmo [22], whereas Syp promotes T2NB entrance into quiescence [21]. Furthermore, the Imp/Syp gradients are essential for the proper progression of early and late temporal transcription factors (TTFs) in the T2NBs [20, 28].

Newborn INPs (nINPs) will mature to become a young INP (yINPs) and continue to age to become a mid INP (mINPs), then old INPs (oINPs). As INPs age, they go through a series of 4–6 divisions, each division resulting in a pair of newborn neurons (nNeurons) or glial cells (Fig. 1B) [2–4, 27, 28]. These neurons will go on to populate the adult *Drosophila* central complex (CX) [27, 32]. The CX, located in the central brain, consists of six neuropils interconnected by different types of neurons and is largely generated from T2NBs (Fig. 1A) [20, 21, 28]. These CX neurons, named for the neuropils they connect, include the two populations of neurons known as E-PGs and P-FRs. There are 35–40 E-PGs with dendrites in the EB and axons in the PB and G, while there are 25–30 PF-Rs with dendrites in the PB and FB and axonal outputs in the RB (Fig. 1C) [20, 27, 28, 32, 34]. E-PGs and PF-Rs are generated from early T2NBs, when Imp expression is high, and oINPs (Fig. 1B) [27]. Importantly, nothing is known about Imp or Syp expression within INP lineages. Here we focus on the expression of Imp and Syp in INPs, and on determining their function in specifying neuronal identity and morphology. We ask: Are Imp and Syp expressed in INPs? Do newborn INPs have the same Imp/Syp levels as their parental NB? Do Imp/Syp form opposing gradients within INP lineages? And lastly, what is the role of Imp in INPs for specifying neuronal identity and morphology?

## Results

### Imp/Syp levels are the same in newborn INPs and T2NBs

**Fig. 2:**
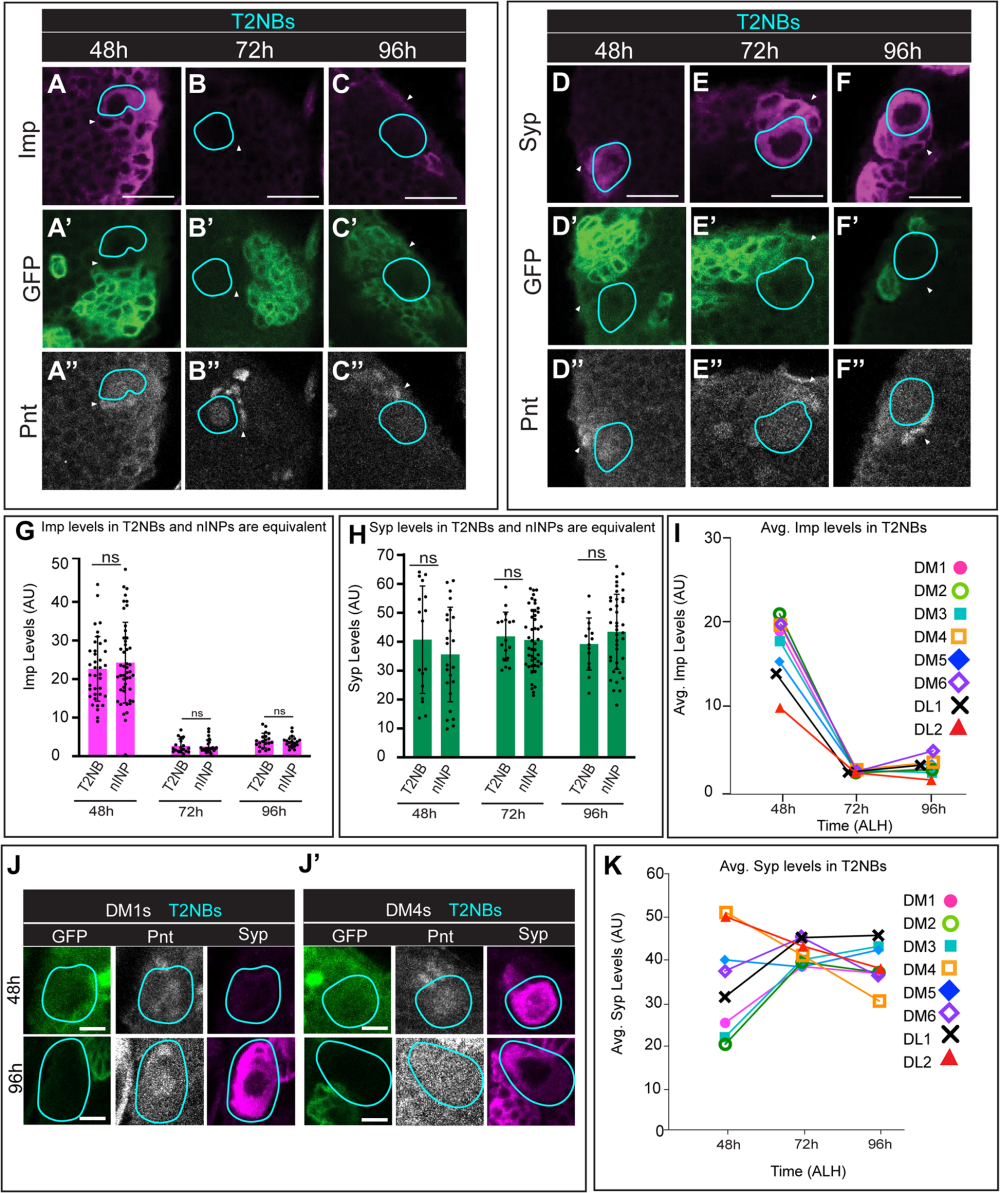
Imp/Syp levels are the same in newborn INPs and T2NBs. **A**-**F** T2NBs (cyan circles; Pnt + GFP-) and nINPs (yellow circles, Pnt + GFP-, contacting T2NBs) at 48 h (**A**), 72 h (**B**), and 96 h (**C**). All timepoints have equivalent Imp (**A**-**C**) and Syp (**D**-**F**) values between T2NBs and nINPs. 12E09 > GFP marks the INP lineage starting at young INP stage. Scale bar 5 μm. **G**-**H** Quantification of Imp (**G**) and Syp (**H**) fluorescent levels in T2NBs and newborn INPs shows no significant differences at 48 h, 72 h, and 96 h. Each point is a single T2NB or nINP, with all 8 T2NBs included *n* = 3–5 brains per timepoint. Student t-tests were used to compare T2NBs and nINPs at each timepoint. *p < 0.05; **p < 0.01; ***p < 0.001; ****p < 0.0001. **I**, **K** Quantification of average Imp (**I**) and Syp (**J**) levels in individual DM1–6 and DL1–2 T2NBs. Note that all T2NBs have a high-low gradient, whereas Syp shows a neuroblast-specific pattern of expression. n = 3–5 brains per timepoint. **J** T2NBs (cyan circles; Pnt + GFP-) at 48 h and 96 h in DM1 (**J**) and DM4 (J’). DM1 expresses Syp in a low-to-high expression gradient at 48 h to 96 h. DM4 Syp expression is the opposite high-to-low expression, similar to Imp. Scale bar 5 μm

We first wanted to know if Imp or Syp expression is present in INPs, and if their initial levels are equivalent to their the parental T2NB at time of INP birth. To target young cells within the INP lineage (nINPs and yINPs) and compare Imp/Syp expression to T2NBs we used 12E09-Gal4 > UAS-GFP. 12E09-Gal4 targets the entire INP lineage starting at yINPs in DM1–6 T2NBs but does not mark parental T2NBs or nINPs. We used Pointed (Pnt) antibody staining, which labels T2NBs, and found that Pnt expression carried over into nINPs, and thus could be used as a marker for those cell types [37] (Fig. 2, Supp. Figure 1). T2NBs are identified as Pnt + GFP-, large size, and location; nINPs are Pnt + GFP- cells in contact with T2NBs (Fig. 2A-F). Imp and Syp fluorescence levels were measured in T2NBs and nINPs at 48 h, 72 h, and 96 h (Fig. 2G, H). Imp levels are high in both T2NBs and nINPs at 48 h, and low in T2NBs and nINPs at 72 h and 96 h (Fig. 2G). At all timepoints there is no difference in Imp or Syp expression between T2NBs and nINPs at any timepoint (Fig. 2G, H;).

Although our focus here is on Imp and Syp in INPs, we collected data on T2NB expression as part of our comparison between T2NB/nINP levels. We confirmed that all 8 T2NBs had a high-to-low Imp gradient (Fig. 2I) as previously reported [20, 28]. Surprisingly, we found NB-specific expression of Syp. We confirm that DL1 has a low-to-high gradient, opposing that of Imp, as previously reported [20], as do DM1–3 (Fig. 2J, K). In contrast, Syp expression in DM5,6 levels stay similar over time, while DM4 and DL2 have an unexpected high-to-low Syp expression gradient (Fig. 2J, K), matching that of Imp (Fig. 2I). We also wanted to measure Syp expression at 24 h, however at this timepoint some T2NBs are still quiescent and are Pnt-, making them only identifiable as either more medial or more lateral. We used Pnt-gal4>UAS-myr::GFP to target proliferative T2NBs and categorized them as either medial or lateral. We were able to see that lateral T2NBs had slightly higher Syp levels at 24 than medial T2NBs (Supp. Figure 2). However, at 24 h Syp levels in T2NBs were universally much lower than 48 h.

We conclude nINPs have the same initial Imp and Syp levels as their parental T2NB. Additionally, we find that that Imp levels in all T2NBs follow a high-to-low temporal gradient, while Syp levels differ across T2NBs, with some co-expressed with Imp in a high-to-low temporal gradient.

### Imp is expressed in a high-to-low gradient in INPs at 48 h

**Fig. 3:**
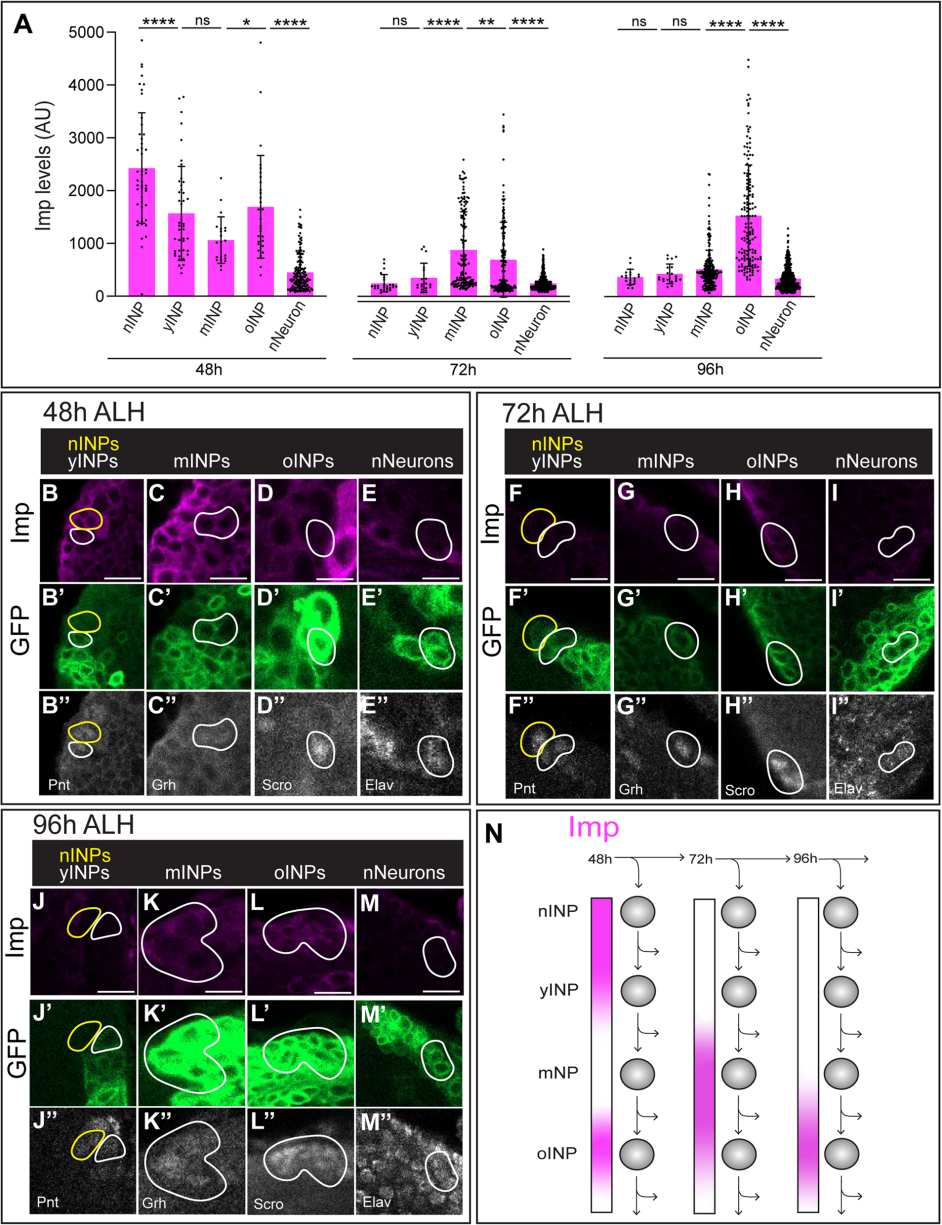
Imp forms a high-low gradient in 48 h INPs. **A** Quantification of Imp fluorescence in nINPs, yINPs, mINPs, oINPs and nNeurons at 48 h, 72 h, and 96 h. Note that Imp forms a high-low gradient in INPs at 48 h; later timepoints show INP age-specific expression. Each point represents a single INP, *n* = 3–5 brains per timepoint. ANOVA analysis was used to compare all cell types at each timepoint. *p < 0.05; **p < 0.01; ***p < 0.001; ****p < 0.0001. **B**-**M** Confocal images of Imp levels in aging INPs at 48 h (**B**-**E**), 72 h (**F**-**I**), and 96 h (**J**-**M**). See Supplemental Fig. 1 for INP staging criteria. 12E09 > GFP marks the INP lineage beginning at yINPs. Scale bar 5 μm. **N** Summary

Having confirmed Imp levels are equivalent in nINPs and T2NBs, we next wanted to know if Imp/Syp expression would follow the same opposing temporal gradients seen in T2NBs [20, 28]. We characterized markers for four stages of INP development, in combination with an early INP driver line (12E09-gal4) or a late INP driver line (16B06-gal4). yINPs are Pnt + GFP+ and border nINPs; mINPs are Grainyhead (Grh) + GFP+; oINPs are Scarecrow (Scro) + GFP+ Elav-; and nNeurons are Elav+ GFP+ Scro- (Fig. 3; summarized in Supp. Figure 1).

We next quantified Imp levels throughout the INP lineage and into nNeurons at 48 h, 72 h, and 96 h (Fig. 3). At 48 h Imp form a high-to-low gradient in the aging INPs, with a slight increase in oINPs (Fig. 3A-E). At 72 h and 96 h Imp is mostly absent (similar to T2NB levels [20, 28], but still shows an uptick of expression in oINPs (Fig. 3F-M). We conclude that in L1 larvae (48 h) Imp is detected in a high-to-low gradient early in the INP lineage, whereas L2 and L3 larvae (72-96 h) have much lower levels of Imp in aging INPs, matching that of T2NBs at those stages, summarized in Fig. 3N.

### Syp forms a high-to-low gradient in aging INPs

**Fig. 4:**
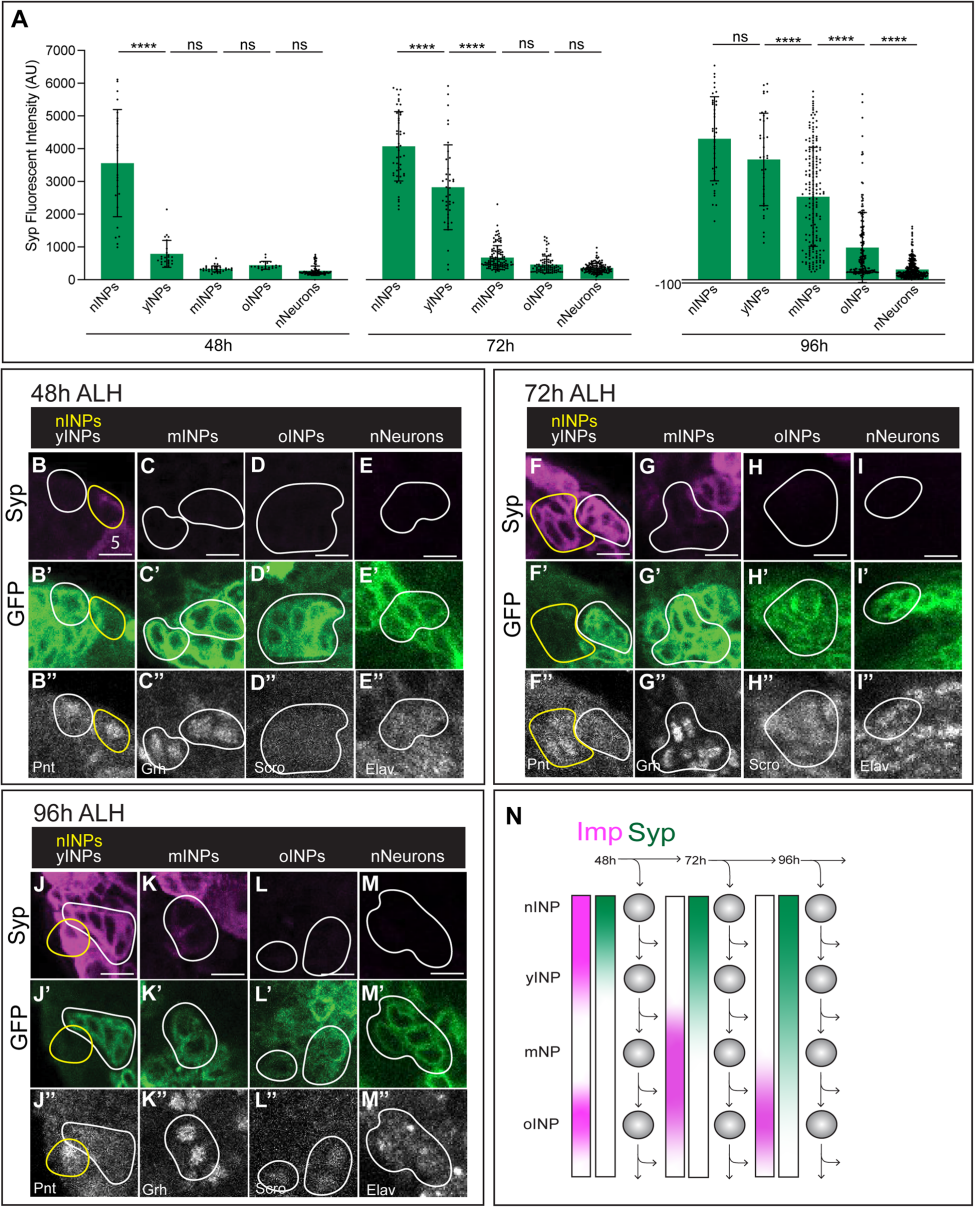
Syp forms a high-low gradient in aging INPs. **A** Quantification of Syp fluorescence in nINPs, yINPs, mINPs, oINPs, and nNeurons at 48 h, 72 h, and 96 h. Syp levels form a high-low gradient in INPs. Each point represents a single INP, *n* = 3–5 brains per timepoint. ANOVA analysis was used to compare all cell types at each timepoint. *p < 0.05; **p < 0.01; ***p < 0.001; ****p < 0.0001. **B**-**M** Confocal images of Syp levels in aging INPs at 48 h (**B**-**E**), 72 h (**F**-**I**), and 96 h (**J**-**M**). See Supplemental Fig. 1 for INP staging criteria. 12E09 > GFP marks the INP lineage beginning at yINPs. Scale bar 5 μm. **N** Summary

We utilized the same genetics and staining methods to quantify Syp expression levels throughout the INP lineage and in nNeurons at 48 h, 72 h, and 96 h. At all three timepoints Syp is detected in a high-to-low gradient (Fig. 4). With the exception of DM4 and DL2, Syp is expressed at higher levels in T2NBs and in newborn INPs at the L2 and L3 larval stages (72-96 h; Fig. 4A, F-M), [20, 28]. Interestingly, we find that both Imp and Syp form high-to-low gradients early in the INP lineage in L1 (48h) larvae; this is in contrast to their robust opposing gradients in T2NBs [20, 28]; summarized in Fig. 4N.

Since Syp expression is T2NB lineage-specific, we wanted to see if this specificity continued into nINPs. We looked at Syp levels in T2NBs and nINPs in each lineage at 48 h to see if Syp expression remained lineage-specific (Supp. Figure 3). We saw that Syp lineage specificity continues into newborn INPs, apart from the DL2 lineage (Supp. Figure 3). Syp levels in the DL2 lineage decrease in newborn INPs, but only to a small extent.

### 16B06-gal > imp^RNAi^ decreases Imp levels in oINPs and nNeurons

Imp function in T2NBs has been previously addressed but its function in INPs remain unclear [20, 28]. To determine the role of Imp specifically in INPs, we initially used the 12E09-gal4 line which is reported to be expressed specifically in INPs [27]. We discovered that 12E09-gal4 was expressed in embryonic T2NBs (Supp. Figure 4), making it unsuitable for INP-specific manipulation of Imp levels. Using 12E09-gal4 to drive Imp^RNAi^ or Imp overexpression (Imp^OE^) generated severe defects in PF-R and E-PG targeting to the CX (Supp. Figure 4C-E), but we were unable to determine if those phenotypes were due to altered Imp levels in the embryonic neuroblast or INP.

**Fig. 5:**
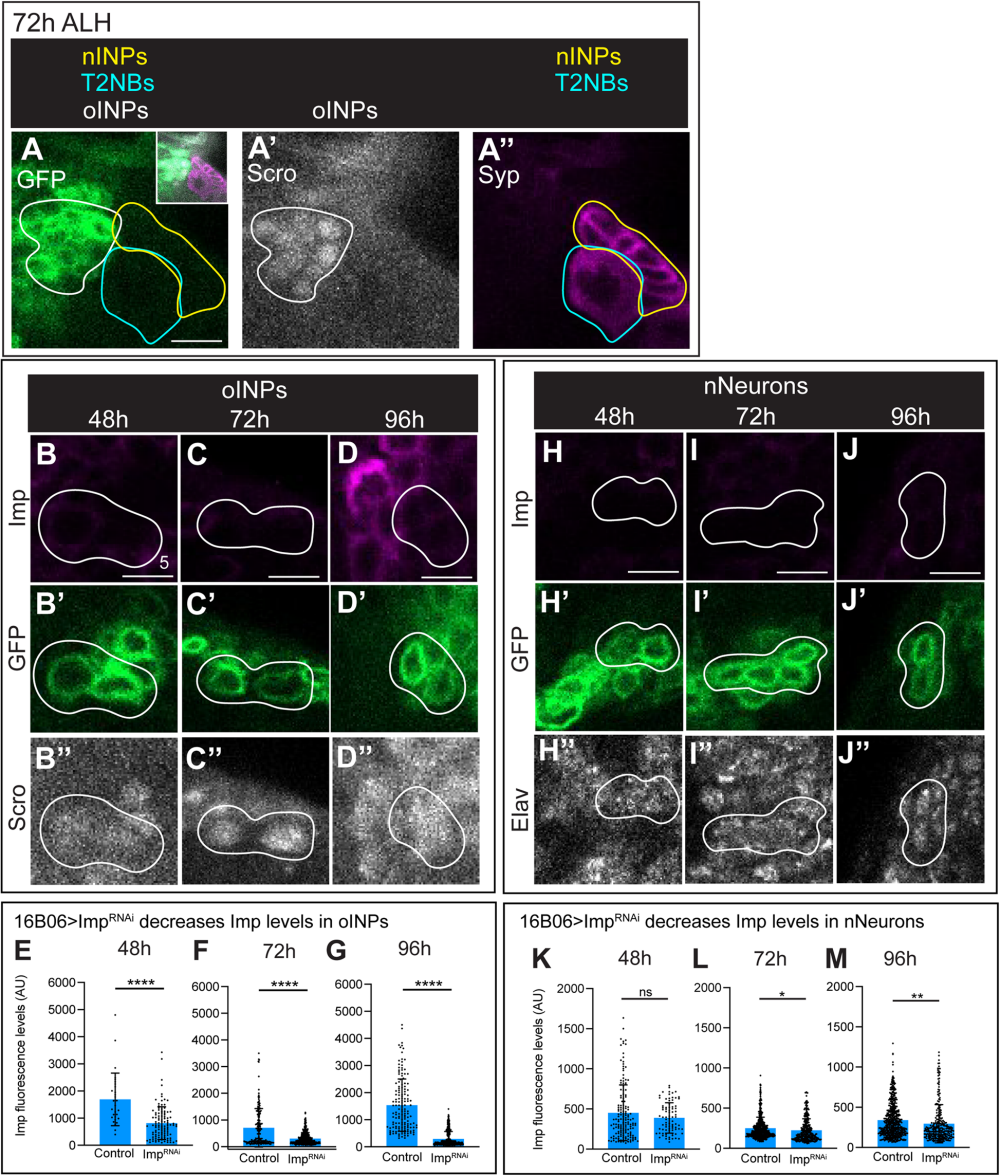
16B06-gal4 > Imp^RNAi^ knocks down Imp in oINPs. **A** 16B06-Gal4 > UAS-GFP UAS-Imp^RNAi^ depletes Imp levels in oINPs, but not T2NBs at 48 h (**B**), 72 h (**C**), and 96 h (**D**). See Supplemental Fig. 1 for INP staging criteria. GFP marks oINPs and nNeurons. Scale bar 5 μm. **B**-**G** Confocal images of Imp levels in oINPs (**B**-**D**), quantified in **E**-**G**. Each point is a single oINPs, *n* = 3–5 brains per timepoint. **H**-**M** Confocal images of Imp levels in nNeurons (**E**-**H**), quantified in **K**-**M**). Each point is a single nNeuron, *n* = 3–5 brains per timepoint. Student t-tests were used to compare Imp levels at each timepoint. *p < 0.05; **p < 0.01; ***p < 0.001; ****p < 0.0001

We next turned to the driver line 16B06-gal4, which we confirm is specifically expressed in oINPs with carryover into nNeurons, with expression continuing into the pupa stages (Fig. 5A). When we used 16B06-gal4 to drive expression of Imp^RNAi^, we observed a decrease in Imp levels in both oINPs and nNeurons at 48 h, 72 h, and 96 h larvae (Fig. 5B-D, H-J, quantified in 5E-G, K-M). In addition, we saw little to no change in oINP cell numbers following any of these manipulations (Supp. Figure 5). We conclude that 16B06-gal4 can be used to specifically reduce Imp levels in oINPs at all stages of larval development, as well as a weaker loss of Imp in nNeurons that is only significant in 72 h and 96 h larvae. From here we chose to focus only on oINPs using 16B06-gal4, due to Imp’s specific increase at the oINP stage.

### 16B06-gal > imp^OE^ decreases imp levels in oINPs, but increases Imp in nNeurons

**Fig. 6:**
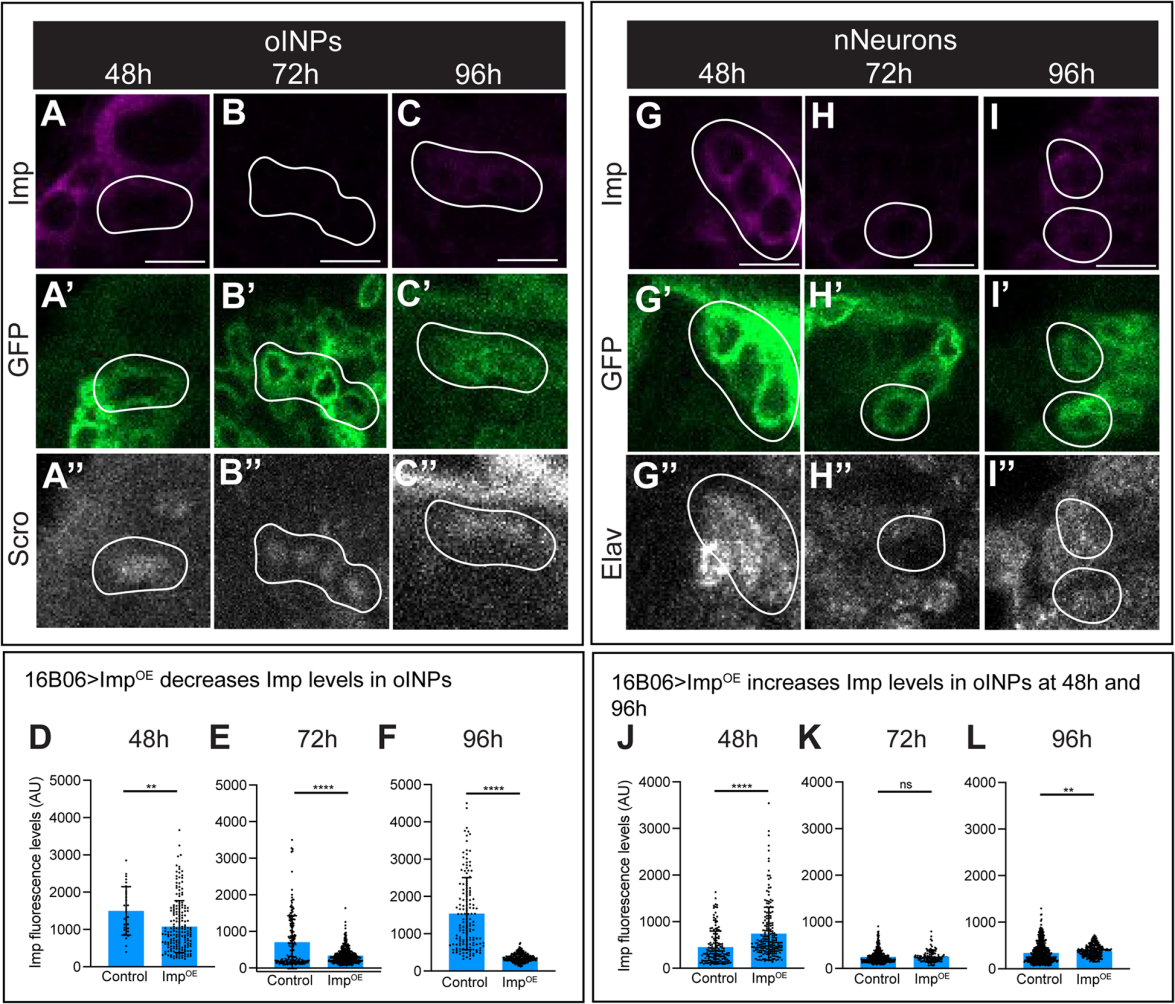
16B06-Gal4 > Imp^OE^ knocks down Imp in oINPs. **A**-**F** 16B06-Gal4 > UAS-GFP UAS-Imp^OE^ depletes Imp levels in oINPs, but not nNeurons at 48 h (**A**), 72 h (**B**), and 96 h (**C**); quantified in **D**-**F**. Each point is a single oINPs, *n* = 3–5 brains per timepoint. **G**-**L** 16B06-Gal4 > UAS-GFP UAS-Imp^OE^ increases Imp levels in nNeurons at 48 h (**G**) and 96 h (**I**), but not at 72 h (**H**); quantified in J-L. Each point is a single oINPs, *n* = 3–5 brains per timepoint. Student t-tests were used to compare Imp levels at each timepoint. *p < 0.05; **p < 0.01; ***p < 0.001; ****p < 0.0001

Next, we wanted to confirm that 16B06-Gal6 > UAS-Imp^OE^ would increase Imp levels. Surprisingly, we found that Imp^OE^ did not increase Imp levels, but counterintuitively decreased Imp levels in oINPs, but caused minor Imp increases in nNeurons (48 h and 96 h) (Fig. 6A-F). In addition, we saw little to no change in oINP cell numbers following Imp^OE^ (Supp. Figure 5). We hypothesize over-expression of Imp may trigger a homeostatic mechanism that reduces Imp levels (see Discussion). Despite the similarity of both Imp^RNAi^ and Imp^OE^ in decreasing Imp levels, we chose to assay both genotypes for neuronal morphology and connectivity defects, where they generated similar yet distinct phenotypes (see below).

### Imp^RNAi^ and Imp^OE^ alter central complex neuropil volume and create ectopic E-PG neuron projections

**Fig. 7:**
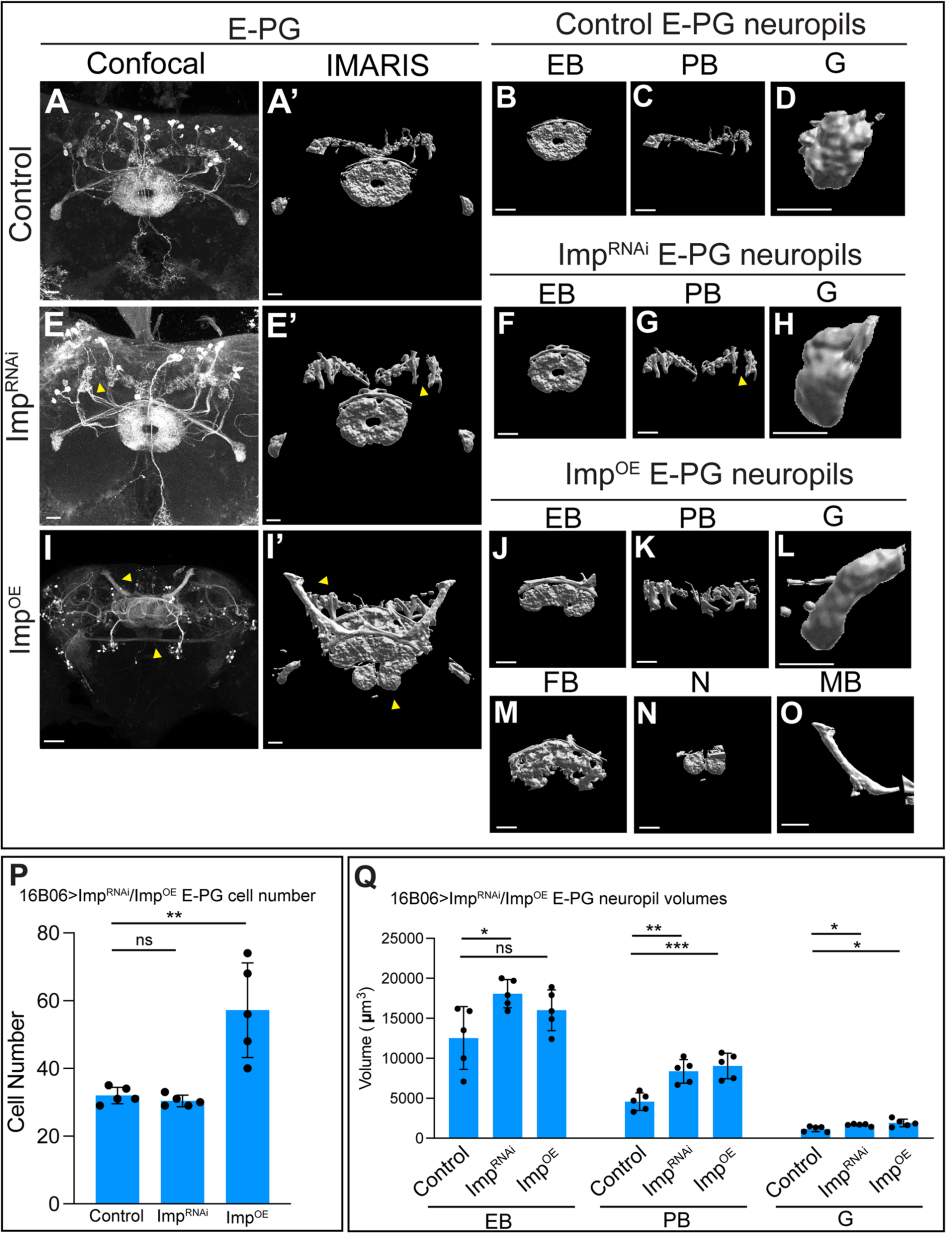
Imp^RNAi^ and Imp^OE^ alter E-PG neuropil targeting. **A**-**D** Control confocal maximum intensity projection of E-PG neurons (**A**) and corresponding IMARIS rendering each targeted neuropil (A’-D). Scale bar 20 μm (**A**-**C**) or 10 μm (**D**). **E**-**H** Imp^RNAi^ confocal maximum intensity projection of E-PG neurons (**E**) and corresponding IMARIS rendering each targeted neuropil (E’-H). Scale bar 20 μm (**E**-**G**) or 10 μm (**H**). **I**-**O** Imp^OE^ confocal maximum intensity projection of E-PG neurons (**I**) and corresponding IMARIS rendering each targeted neuropil (I′-O); note that Imp^OE^ results in E-PG neurons generating ectopic projections to the fan-shaped body (FB), noduli (**N**), and mushroom body (MB). Scale bar 20 μm (**J**, **K**, **M**, **N**) or 10 μm (L, O). **P**, **Q** Quantification of cell numbers (**P**), and neuropil volume (**Q**); each point represents an adult *Drosophila* brain, *n* = 3–5 brains in control, Imp^RNAi^, and Imp^OE^. Student t-tests were used to compare cell numbers to control. ANOVA analysis was used to compare neuropil volumes back to control. *p < 0.05; **p < 0.01; ***p < 0.001; ****p < 0.0001

**Fig. 8:**
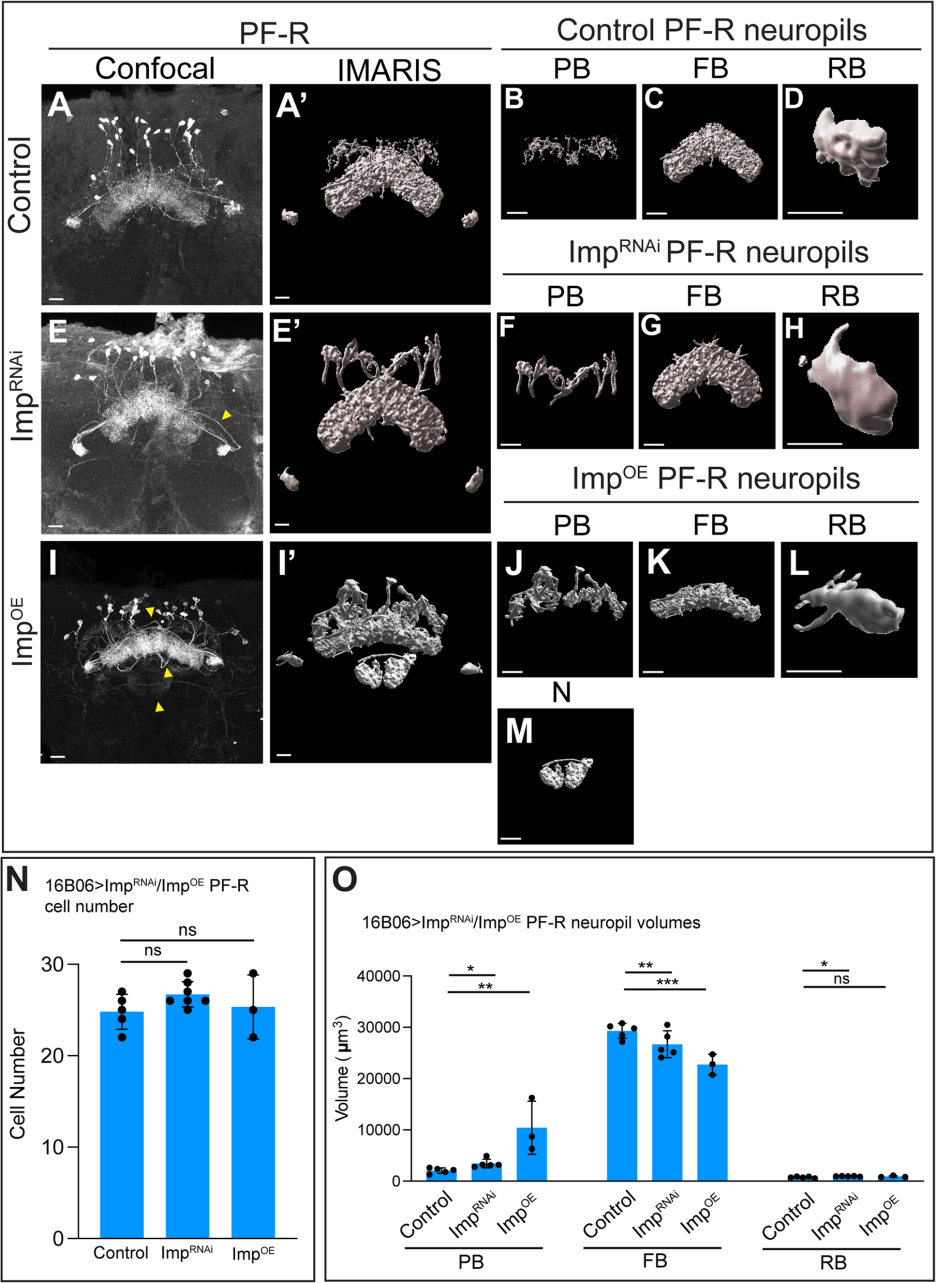
Imp^RNAi^ and Imp^OE^ disrupts PF-R neuropil targeting but not cell number. **A**-**D** Control confocal maximum intensity projection of PF-R neurons (**A**) and corresponding IMARIS rendering each targeted neuropil (A’-D). Scale bar 20 μm (**A**-**C**) or 10 μm (**D**). **E**-**H** Imp^RNAi^ confocal maximum intensity projection of PF-R neurons (**E**) and corresponding IMARIS rendering each targeted neuropil (E’-H). Scale bar 20 μm (**E**-**G**) or 10 μm (**H**). **I**-**O** Imp^OE^ confocal maximum intensity projection of PF-R neurons (**I**) and corresponding IMARIS rendering each targeted neuropil (I′-M); note that Imp^OE^ results in PF-R neurons generating ectopic projections to the noduli (**N**). Scale bar 20 μm (I-K, M) 10 μm (**L**). **N**, **O** Quantification of cell numbers (**N**), and neuropil volume (**O**); each point represents an adult *Drosophila* brain, *n* = 3–5 brains in control, Imp^RNAi^, and Imp^OE^. Student t-tests were used to compare cell numbers to control. ANOVA analysis was used to compare neuropil volumes back to control. *p < 0.05; **p < 0.01; ***p < 0.001; ****p < 0.0001

To decipher the role of Imp in oINPs and nNeurons in regulating neuronal morphology, we used 16B06-Gal4 > UAS-Imp^RNAi^ or UAS-Imp^OE^ to alter Imp levels and assayed two neuron populations that are derived from old INPs [27]: E-PG neurons (this section; Fig. 7) and PF-R neurons (following section; Fig. 8).

We used the LexA/LexAop system to visualize adult brain E-PG neurons (60D05-LexA>LexAop-GFP or tdTomato). In controls, E-PG neurons innervate the EB, PB, and G neuropils, shown as a confocal image (Fig. 7A) and Imaris renderings of each individual neuropil (Fig. 7A-D‘). Quantification of cell number and neuropil volumes is shown in Fig. 7P-Q. In contrast, Imp^RNAi^ resulted in an enlargement of all three neuropils (Fig. 7E-H; quantified in 7Q), without altering E-PG neuron numbers (Fig. 7P). Imp^OE^ had a similar phenotype with enlarged EB, PB, and Gall neuropils (Fig. 7I-L), but differed in exhibiting inappropriate projections into the FB, N, and mushroom body (Fig. 7I, M-O); the latter normally not innervated by T2NB progeny. There was also a large increase in E-PG neuron numbers (Fig. 7P); the relationship of increased neuron numbers and ectopic neuropil targeting is unknown; see Discussion. We conclude that Imp acts in INPs or newborn neurons to promote proper E-PG neuropil targeting within the CX. Differences between Imp^RNAi^ and Imp^OE^ phenotypes may be due to different decreases in Imp levels, or potentially due to transient increases in Imp levels prior to homeostatic reduction in Imp levels.

### Imp^RNAi^ and Imp^OE^ alter central complex neuropil volume and create PF-R neuron mistargeting

We next wanted to see if these results were consistent in PF-Rs, which are also derived from oINPs [27]. We used the LexA/LexAop system to visualize adult brain P-FR neurons (37G12-LexA>LexAop-GFP or tdTomato). In controls, PF-R neurons innervate the PB, FB, and RB neuropils, shown as a confocal image (Fig. 8A) and Imaris renderings of each individual neuropil (Fig. 8A‘-D). Quantification of cell number and neuropil volumes is shown in Fig. 8N-O. In contrast, Imp^RNAi^ resulted in varying alterations in the volume of each neuropil: the PB and RB were enlarged, while the FB was reduced (Fig. 8E-H; quantified in 8O), without altering PF-R neuron numbers (Fig. 8P). Imp^OE^ had a similar phenotype as Imp^RNAi^ in having enlarged EB and reduced FB neuropils (Fig. 8I-L) but differed in exhibiting inappropriate projections into the Noduli (Fig. 8M). There were no increases in PF-R neuron numbers (Fig. 8N). We conclude that Imp acts in INPs or newborn neurons to promote proper PF-R neuropil targeting within the CX.

## Discussion

Previous research has documented the opposing Imp/Syp gradients in both type 1 and 2 NBs [16, 20, 27, 28]. However, Imp/Syp expression and function in INPs has not been characterized. Here we show that Imp/Syp expression in T2NBs is the same as newborn INPs. This finding is consistent across all 8 T2NB lineages, no matter the level of Imp/Syp expression. Additionally, we confirmed that all T2NBs express the high-to-low temporal Imp gradient, extending previous work that focused on DL1/DL2 T2NBs, type 1 mushroom body and antennal lobe NB lineages [20]. Unlike in mushroom body and antennal lobe NBs [16], Syp expression in T2NBs is not always expressed in a low-to-high gradient. Instead, only DL1 and DM1–3 exhibit this familiar Syp gradient, while DM4 and DL2 display an opposite high-to-low expression gradient, more similar to Imp. Separately, DM5 and DM6 each have unique Syp expression pattern: in DM5, Syp remains relatively even at a consistent level throughout larval development; in DM6, Syp peaks at 72 h and decreases to a lower level at 48 h and 96 h. While previous work has described the Imp’s role in Syp inhibition [16, 20, 22, 28], we conclude that additional factors must be regulating Syp expression to allow it to overlap with Imp expression. One hypothesis is the increased levels of Syp in DM4 and DL2 are somehow uncoupled from Imp, preventing Syp-mediated Imp down regulation. An alternate hypothesis stems from the following findings, showing that mammalian SYNCRIP/hnRNPQ (homologous to *Drosophila* Syp) [18] expression can be promoted through long non-coding RNA (lnRNA) NT5E [36]. LnRNA NT5E promotes cell proliferation in human pancreatic cancer cell samples during epithelial-mesenchymal transition in vitro [36]. The lnRNA N5TE genomic location is close to the Syp locus, and activation of lnRNA NT5E results in Syp activation in vitro [36]*.* T2NB lineage-dependent gene expression of lnRNAs located close to Syp could be the cause the variation in Syp levels in each T2NB. Lastly, previous work has shown that co-expression of Imp and Syp in mushroom body NBs (MBNBs) results in a neuronal identity (α’β’ neurons) distinct from neurons produced with only high Imp (γ neurons) or high Syp levels (αβ neurons) [16]. Thus, independent of the mechanism resulting in Imp and Syp overlap, the Imp/Syp co-expression we report in INP lineages may be necessary to specify distinct types of neurons.

At 24 h some T2NBs are still in quiescence and T2NBs can only be differentiated at more medial or lateral instead of their specific lineages. At 24 h Syp expression is low in medial T2NBs with only a slight increase in lateral T2NBs. Syp levels don’t seem to become lineage-specific until 48 h into neurogenesis. This further supports the hypothesis of an independent mechanism for Imp and Syp overlap in specific T2NB lineages.

After confirming Imp/Syp expression patterns in T2NBs and validating that Imp/Syp levels are the same in newborn INPs, we found that at 48 h, INPs formed a high-to-low Imp gradient. In contrast, at 72 h and 96 h, Imp showed a peak of expression in old INPs (Fig. 3A). This brief increase could be due to regulation of Imp by Lin-28, another RNA-binding protein expressed early in T2NB lineages. In *Drosophila* intestinal stem cells, (ISCs) Lin-28 and Imp are both expressed to promote ISC proliferation and regulation of each other [25]. In fact, overexpression of Lin-28 resulted in an increase of Imp expression [20, 25, 28]. The old INP Imp peak could also be playing a role in generating gene expression differences between old INPs from young INPs [2]. For example, young INPs express the transcription factor Dichaete (D) and are negative for the transcription factor Eyeless (Ey), whereas oINPs are the opposite, D- and Ey + [2]. Another striking difference between young and old INPs is the generation of different types of CX neurons. Young INPs generate P-FN neurons [27, 32, 34], whereas old INPs generate PF-R and E-PG neurons [27]. The Imp expression peak in old INPs could help distinguish these neuronal identities.

Imp^RNAi^ was able to significantly reduce Imp levels in INPs across all timepoints, with the exception of newborn neurons at 48 h ((Fig. 5E-G, K-M). Surprisingly, Imp^OE^ also significantly decreased Imp levels in INP lineages (Fig. 6D-F, J-L). We hypothesize that this unexpected drop in Imp levels following Imp^OE^ is caused by tight a homeostatic regulation of Imp levels. For example, the microRNA (miRNA) *let-7* inhibits Imp in the *Drosophila* testis stem cell niche [29]; if Imp promotes *let-7* expression it could produce a negative feedback loop that keeps Imp levels low. Traditionally miRNAs bind the 3′ UTRs of mRNA targets, but the Imp^OE^ used in this work lacked its normal 3′ UTR. Surprisingly, previous work shows that regulation of *let-7* in the 5′ UTR does occur [17]. Complimentary binding sites from the 3′ UTR of *let-7* were added to the 5′ UTR of its mRNA target *lin-41* from *C. elegans* [17]. When transfected into mammalian cells with endogenous *let-7*, it was sufficient for *lin-41* repression [17]. Whether *let-7* or another factor is activated by Imp and then represses Imp levels remains to be determined.

Expression of 16B06-Gal4 was undetectable in T2NBs, which was a prerequisite to characterizing Imp in Imp^RNAi^ or Imp^OE^ specifically in INPs. However, this driver was also expressed in newborn neurons, thus limiting our ability to distinguish Imp function in INPs versus neurons. Previous work supports a role for Imp in postmitotic neurons. The mushroom body is made of up three types of neurons: γ, α’β’, and αβ neurons [16]. Previous research has shown that Imp forms mobile ribonucleoprotein (RNP) granules that are transported to γ axons [31]. A mutated form of Imp lacking its prion-like domain (PLD) caused a change in axon morphology through Imp-dependent remodeling [31]. ImpΔPLD caused a decrease in γ neuron axon length, and loss of axonal branching [31]. This raises the possibility that altered Imp levels in newborn neurons in INP lineages may result in morphological defects.

Imp^OE^ only causes an increase in Imp levels in newborn neurons (Fig. 6J, L), however this increase in Imp expression does not change the number of newborn neurons (Supp. Figure 5B), despite causing an increase in E-PG cell numbers in the adult brain (Fig. 7P). The increase in E-PG number could be due to the role of Imp in regulating apoptosis. The mammalian paralogue of Imp, IMP-3, prevents cell death after misexpression in lymphoblast cells [15], and inhibits apoptosis in human colorectal cancer cells [8]. We hypothesize that the increased levels of Imp seen in Imp^OE^ in newborn neurons at 48 h and 96 h (Fig. 6J, L) could account for the increased E-PG numbers in adults.

Whereas Imp only forms a high-to-low gradient in INPs at 48 h, Syp consistently forms a high-to-low gradient in the INP lineages at all timepoints assayed, with the Syp gradient extending longer into the INP lineage at later development stages (Fig. 4A). This high-to-low Syp gradient in INPs is opposite the low-to-high Syp gradient seen in mushroom body and antennal lobe NB lineages. This is surprising, as Syp is known to promote differentiation in other progenitors. The role of high Syp in young, proliferating INPs is unknown. Perhaps high Syp is required for limiting INP proliferation to 4–6 cell divisions. In addition, the co-expression of Imp and Syp in young INPs may result distinct neuronal identities that are specified by the combination of Imp and Syp, similar to the α’β’neurons in the mushroom body NB lineages [16].

## Materials and methods

### Antibodies and immunostaining

All larvae and adult *Drosophila* were raised at 25 °C and dissected in Hemolymph Like buffer 3.1 (HL3.1) (NaCl 70 mM, KCl 5 mM, CaCl_2_ 1.5 mM, MgCl_2_ 4 mM, sucrose 115 mM, HEPES 5 mM, NaHCO_3_ 10 mM, and Trehalose 5 mM in double distilled water). Larvae were grown to specified time points, dissected, mounted on poly-D-lysine coated slips (Neuvitro, Camas, WA), and incubated for 30 minutes (adults incubated for 40 m) in 4% paraformaldehyde solution in Phosphate Buffered Saline (PBS) with 1% Triton-X (1% PBS-T) at room temperature. Larval brains were washed twice with 0.5% PBS-T (adults brains were washed twice with 1%PBS-T) and incubated for 2–4 days (adult brains were incubated for 4–10 days) at 4 °C in a blocking solution of 1% goat serum (Jackson ImmunoResearch, West Grove, PA), 1% donkey serum (Jackson ImmunoResearch, West Grove, PA), 2% dimethyl sulfoxide in organosulfur (DMSO), and 0.003% bovine serum albumin (BSA) (Fisher BioReagents, Fair Lawn, NJ Lot #196941). Larval brains were incubated for two nights at 4 °C in a solution of primary antibodies (see Table 1) in 0.5% PBS-T. Adult brains were incubated overnight at 4 °C in a solution of primary antibodies (see Table 1) in 1% PBS-T. Larval and adult brains were washed for at least 30 minutes in 0.5% PBS-T (adults in 1% PBS-T) at room temperature, and then incubated overnight at 4 °C in a solution of secondary antibodies (see Table 1) in 0.5% PBS-T (adults in 1% PBS-T). Larval brains were washed in 0.5% PBS-T (adults in 1% PBS-T) for at least 30 minutes at room temperature. Larval brains were dehydrated by going through a series of 10-minute washes in 30, 50, 70, and 90% EtOH, and two rounds of 10 minutes in 100% EtOH and two rounds of 10 minutes in xylene (MP Biomedicals, LLC, Saolon, OH, Lot# S0170. Adult brains were dehydrated by going through a series of 12-minute washes in 30, 50, and 70% EtOH, 15 minutes in 90% EtOH, and two rounds of 18 minutes in 100% EtOH and two rounds of 18 minutes in xylene. Both larval and adult brains were mounted in dibutyl phthalate in xylene (DPX; Sigma-Aldrich, cat. no. 06522). Larval brains sat in DPX for at least 48 hours (72 h for adult brains) at room temperature before being imaged or stored at 4 °C. Staining combinations for T2NB lineage and INP stage identification can be seen in Table 2.

**Table 1:** Key Resource Table

**Reagent**	**Designation**	**Source**	**Identifiers**	**Additional information**
Species (*D melanogaster*)	12E09-Gal4, 10xUAS-mCD8::GFP	Doe lab	n/a	Early INP driver
Species (*D melanogaster*)	16B06-Gal4, 10xUAS-mCD8::GFP	Doe lab	n/a	Late INP driver
Species (*D melanogaster*)	UAS-ImpRNAi	BDSC	#34977	Imp knockdown
Species (*D melanogaster*)	UAS-Imp	MacDonald lab (UT Austin)	n/a	Imp overexpression
Species (*D melanogaster*)	UAS-myr::GFP	BDSC	#32198	Membrane bound GFP under UAS control
Antibody	Chicken anti-GFP	Abcam (Eugene, OR)	n/a	1:1000
Antibody	Rabbit anti-Imp	MacDonald lab (UT Austin)	n/a	1:1000
Antibody	Rabbit anti-Syp	Genescript (Piscataway, NJ)	#4060	1:1000
Antibody	Rat anti-Dpn	Abcam (Eugene, OR)	n/a	1:20
Antibody	Rat anti-Grh	Desplan lab (NYU)	n/a	1:500
Antibody	Guinea pig anti-Scro	Genescript (Piscataway, NJ)	#7153	1:200
Antibody	Mouse anti-Elav	DSHB (Iowa City, IA)	9F8A9-CM	1:100
Antibody	Guinea pig anti-Pnt	Genescript (Piscataway, NJ)	#P0111	1:500
Antibody	Rabbit anti-DsRed	Rockland (Pottstown, PA)	#48710	1:500
Antibody, polyclonal	Secondary antibodies	Thermofisher (Eugene, OR)	n/a	1:200 or 1:400 (Dpn and Scro only)
**Fly genotypes used in each figure**			**Figure**	**Synopsis**
; 37G12-LexA; 13xLexAop-myr::GFP;			Figure 1C	PF-R labeling
; 60D05-LexA; 13xLexAop-myr::GFP;			Figure 1C	E-PG labeling
;; 12E09-Gal4, 10xUAS-mCD8::GFP;			Figure 2A, B	INP lineage labeling
;; 12E09-Gal4, 10xUAS-mCD8::GFP;			Figure 3B-M	INP lineage labeling
;; 12E09-Gal4, 10xUAS-mCD8::GFP;			Figure 4B-M	INP lineage labeling
;; 16B06-Gal4, 10xUAS-myr::GFP;			Figure 5A	oINP and nNeuron labeling
;; 16B06-Gal4, 10xUAS-myr::GFP; x;; UAS-Imp^RNAi^			Figure 5B-J	oINP Imp^RNAi^ and labeling
;; 16B06-Gal4, 10xUAS-myr::GFP; x;; UAS-Imp^OE^			Figure 6A-I	oINP Imp^OE^ and labeling
; 60D05-LexA; x; 13xLexAop-myr::GFP; 16B06-Gal4			Figure 7A-D	Control E-PG neurons
; 60D05-Gal4; UAS-mCherry^RNAi^ x; 13xLexAop-myr::GFP; 16B06-Gal4			Not shown	Control E-PG neurons
; UAS-mCherry; 60D05-LexA x; 13xLexAop-myr::GFP; 16B06-Gal4			Not shown	Control E-PG neurons
; 13xLexAop-myr::GFP; UAS-ImpRNAi x; 60D05-LexA; 16B06-Gal4			Figure 7E-H	oINP Imp^RNAi^, E-PG labeling
; UAS-Imp; 20xLexAop-DsRed x; 60D05-LexA; 16B06-Gal4			Figure 7I-O	oINP Imp^OE^, E-PG labeling
; 37G12-LexA; x; 13xLexAop-myr::GFP; 16B06-Gal4			Figure 8A-D	Control PF-R neurons
; 37G12-Gal4; UAS-mCherry^RNAi^ x; 13xLexAop-myr::GFP; 16B06-Gal4			Not shown	Control PF-R neurons
; UAS-mCherry; 60D05-LexA x; 13xLexAop-myr::GFP; 16B06-Gal4			Not shown	Control PF-R neurons
; 13xLexAop-myr::GFP; UAS-ImpRNAi x; 37G12-LexA; 16B06-Gal4			Figure 7E-H	oINP Imp^RNAi^, PF-R labeling
; UAS-Imp; 20xLexAop-DsRed x; 37G12-LexA; 16B06-Gal4			Figure 7I-M	oINP Imp^OE^, PF-R labeling

**Table 2:** Cell Type Identification Markers

Cell type	Identification
T2NB	GFP- Pnt+, location within brain *note that DL1 and DL2 are not labeled with 12E09-Gal4, they were found using the same method.
nINP	GFP- Pnt+, bordering T2NBs
yINP	GFP+ Pnt, bordering nINPs
mINP	GFP+ Grh + Scro-
oINP	GFP+ Grh- Scro+
nNeuron	GFP+ Scro- Elav+

### Imaging and statistical analysis

All Imp data was collected with identical confocal settings; all Syp data were collected with identical confocal settings. Fluorescent images were collected on Zeiss LSM 800. Cells were counted using the cell counter plugin in FIJI (https://imagej.net/software/fiji/). Imp/Syp pixel density in each cell type was calculated in FIJI. In FIJI, cells were manually selected in a 2D plane at the largest cross section of the cell with the polygon lasso tool, and the area and Raw Integrated Density (RID) was measured. The nucleus of each cell went through the same analysis steps. Imp is cytoplasmic and measuring fluorescence in the nucleus functioned as background subtraction. Imp levels were normalized to cell area using the equation: (Cell Body^RID^ – Nucleus^RID^) / (Cell Body^Area^ – Nucleus^Area^). ANOVA or two-tailed student t-tests were used to compare two sets of data. *p < 0.05; **p < 0.01; ***p < 0.001; ****p < 0.0001. All graphs and statistical analysis were done in Prism (GraphPad Software, San Diego, CA). Note that we were unable to quantify Imp fluorescence in mitotic cells.

In adult brains, morphology analysis and neuropil volume quantifications for E-PG and PF-R neurons were completed in IMARIS (Oxford Instruments, imaris.oxinst.com). The surfaces tool was used to select individual neuropils.

### Figure production

Images for figures were taken in FIJI. Figures were assembled in Adobe Illustrator (Adobe, San Jose, CA). Any changes in brightness or contrast were applied to the entire image.

### Supplementary Information

**Additional file 1: Supplementary Video 1. **Imaris surface reconstruction of larval central brain at 60h. Magenta spheres represent T2NBs and their location within the larval brain. Scale bar 20 μm.

**Additional file 2 : Supplemental Fig. 1.** INP staging criteria. Schematic showing markers that define different stages in INP lineage progression. T2NBs (green, GFP- Pnt+); nINPs contact the parental NB (purple, GFP- Pnt+); yINPs (yellow, GFP+ Pnt+) border nINPs; mINPs (blue, GFP+ Grh + Scro-); oINPs (pink, GFP+ Grh- Scro+); and nNeurons (orange, GFP+ Elav+ Scro-). GFP was driven in nINPs, yINPs, mINPs and oINPs with 12E09-Gal4, and in oINPs and nNeurons with 16B06-Gal4. **Supplemental Fig. 2.** At 24 h T2NB lineages can only be characterized as medial and lateral. (A) 12E09 > UAS-GFP at 24 h targets proliferative T2NBs (GFP+, Pnt + yellow circles). Scale bar 5 μm. (B) Quantification of Syp levels in medial and lateral T2NBs at 24 h. *n* = 5 brains. Student t-tests were used to compare medial cells to lateral cells. *p < 0.05; **p < 0.01; ***p < 0.001; ****p < 0.0001. **Supplemental Fig. 3.** Lineage specific Syp levels in T2NBs and nINPs is equivalent except for DL2. (A) Quantification of Syp levels in T2NBs and nINPs in each specific lineage. *n* = 5 brains. Student t-tests were used to compare medial cells to lateral cells. *p < 0.05; **p < 0.01; ***p < 0.001; ****p < 0.0001. **Supplemental Fig. 4.** 12E09-Gal4 is expressed in embryonic T2NBs and is required for PF-R and E-PG neuron morphology. (A) 12E09-Gal4 > UAS-GFP in embryonic T2NBs. T2NBs (GFP+ Pnt+, cyan circles). Scale bar 5 μm. (B) Schematic of 12E09-Gal4 expression in embryonic and larval T2NBs and n/yINPs. (C) 12E09-Gal4 > UAS-Imp^RNAi^ turns on earlier in development. T2NBs (cyan circles, GFP- Dpn+), nINPs (yellow circles, GFP- Dpn+), and yINPs (white circles, GFP+ Dpn-) show a loss of Imp at 48 h in T2NBs. Scale bar 5 μm. (D-E) Confocal maximum intensity projections of control, Imp^RNAi^ and Imp^OE^ in PF-R and E-PG neurons. *n* = 5, scale bar 20 μm. **Supplemental Fig. 5.** 16B06 > Imp^RNAi^ causes an increase in cell number at 48 h and 72 h. (A-B) Number of oINPs (A) and nNeurons (B) in control, Imp^RNAi^ and Imp^OE^. Each point is an oINP (A) or nNeuron (B). *n* = 3–5 brains. Student t-tests were used to compare cell numbers to control. *p < 0.05; **p < 0.01; ***p < 0.001; ****p < 0.0001.

## Data Availability

No new data sets or fly genotypes were developed.
