# Supplementary figures and images for: Imp is expressed in INPs and newborn neurons where it regulates neuropil targeting in the central complex
Source: Neural Dev. 2023 Nov 29;18:9. doi: 10.1186/s13064-023-00177-9 (PMC10685609; doi:10.1186/s13064-023-00177-9)

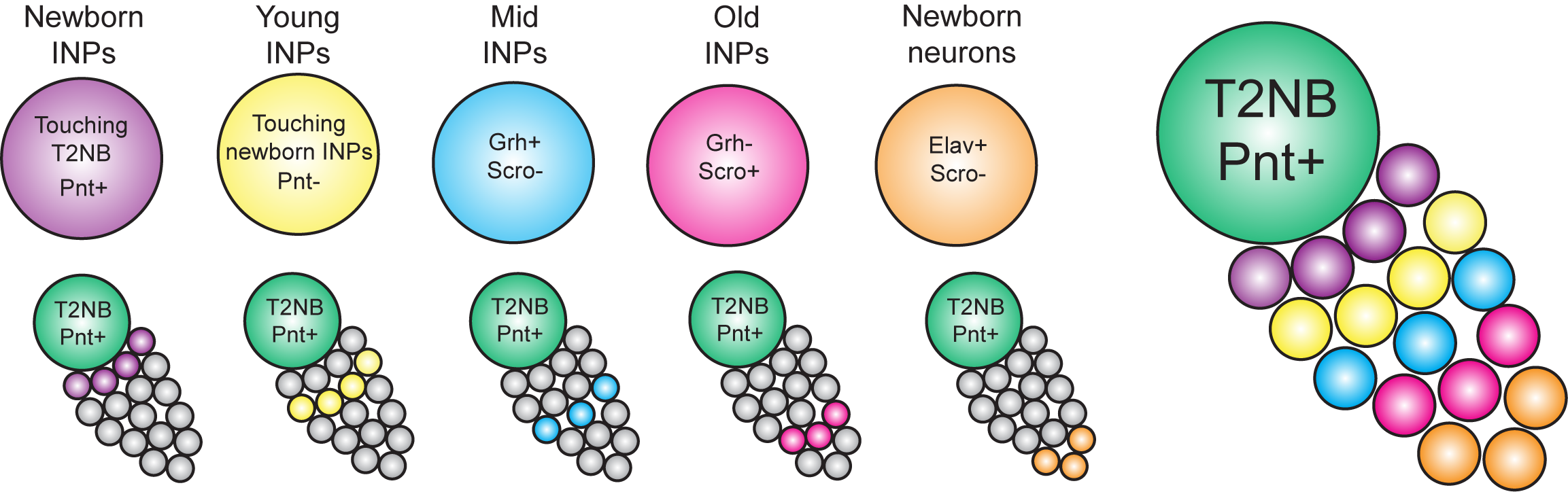

Supplement: Supplementary file 2 — Additional file 2 : Supplemental Fig. 1. INP staging criteria. Schematic showing markers that define different stages in INP lineage progression. T2NBs (green, GFP- Pnt+); nINPs contact the parental NB (purple, GFP- Pnt+); yINPs (yellow, GFP+ Pnt+) border nINPs; mINPs (blue, GFP+ Grh + Scro-); oINPs (pink, GFP+ Grh- Scro+); and nNeurons (orange, GFP+ Elav+ Scro-). GFP was driven in nINPs, yINPs, mINPs and oINPs with 12E09-Gal4, and in oINPs and nNeurons with 16B06-Gal4. Supplemental Fig. 2. At 24 h T2NB lineages can only be characterized as medial and lateral. (A) 12E09 > UAS-GFP at 24 h targets proliferative T2NBs (GFP+, Pnt + yellow circles). Scale bar 5 μm. (B) Quantification of Syp levels in medial and lateral T2NBs at 24 h. n = 5 brains. Student t-tests were used to compare medial cells to lateral cells. *p < 0.05; **p < 0.01; ***p < 0.001; ****p < 0.0001. Supplemental Fig. 3. Lineage specific Syp levels in T2NBs and nINPs is equivalent except for DL2. (A) Quantification of Syp levels in T2NBs and nINPs in each specific lineage. n = 5 brains. Student t-tests were used to compare medial cells to lateral cells. *p < 0.05; **p < 0.01; ***p < 0.001; ****p < 0.0001. Supplemental Fig. 4. 12E09-Gal4 is expressed in embryonic T2NBs and is required for PF-R and E-PG neuron morphology. (A) 12E09-Gal4 > UAS-GFP in embryonic T2NBs. T2NBs (GFP+ Pnt+, cyan circles). Scale bar 5 μm. (B) Schematic of 12E09-Gal4 expression in embryonic and larval T2NBs and n/yINPs. (C) 12E09-Gal4 > UAS-ImpRNAi turns on earlier in development. T2NBs (cyan circles, GFP- Dpn+), nINPs (yellow circles, GFP- Dpn+), and yINPs (white circles, GFP+ Dpn-) show a loss of Imp at 48 h in T2NBs. Scale bar 5 μm. (D-E) Confocal maximum intensity projections of control, ImpRNAi and ImpOE in PF-R and E-PG neurons. n = 5, scale bar 20 μm. Supplemental Fig. 5. 16B06 > ImpRNAi causes an increase in cell number at 48 h and 72 h. (A-B) Number of oINPs (A) and nNeurons (B) in control, ImpRNAi and ImpOE. Each point is [file 13064_2023_177_MOESM2_ESM.zip › fig sup 1.tif]

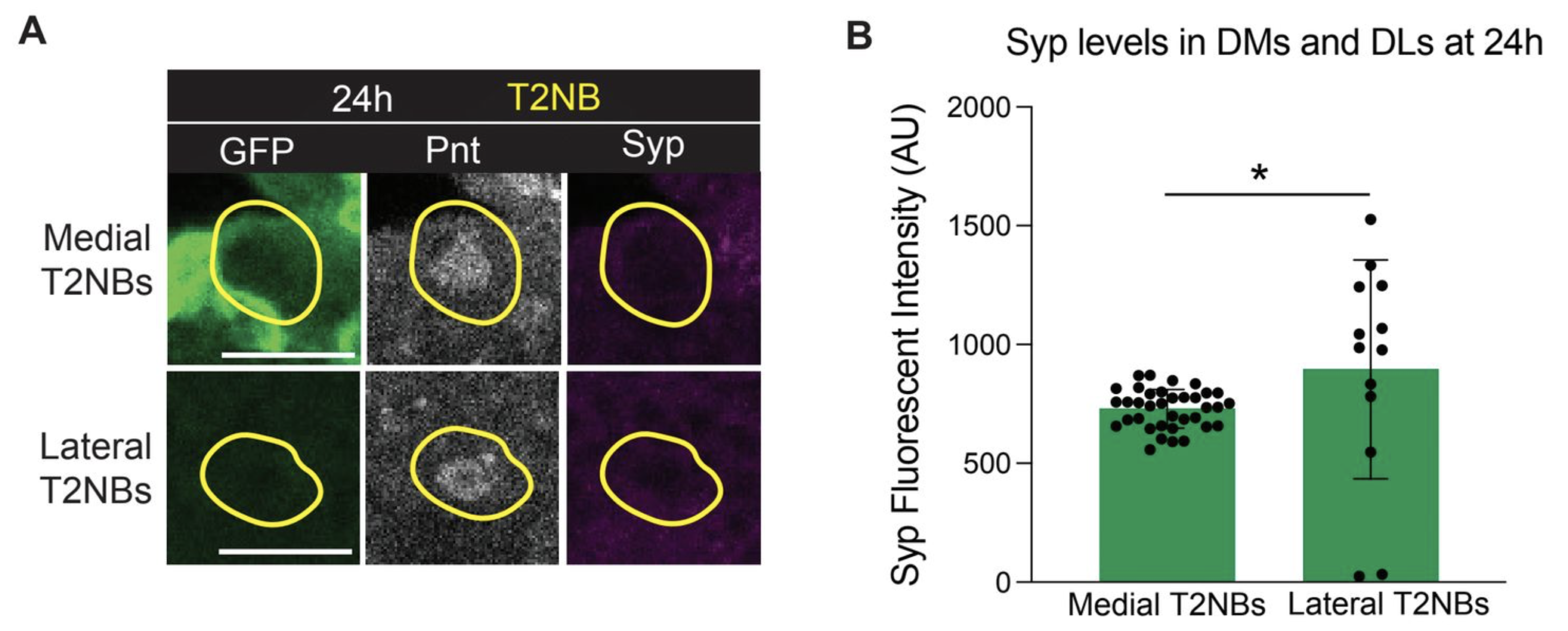

Supplement: Supplementary file 2 — Additional file 2 : Supplemental Fig. 1. INP staging criteria. Schematic showing markers that define different stages in INP lineage progression. T2NBs (green, GFP- Pnt+); nINPs contact the parental NB (purple, GFP- Pnt+); yINPs (yellow, GFP+ Pnt+) border nINPs; mINPs (blue, GFP+ Grh + Scro-); oINPs (pink, GFP+ Grh- Scro+); and nNeurons (orange, GFP+ Elav+ Scro-). GFP was driven in nINPs, yINPs, mINPs and oINPs with 12E09-Gal4, and in oINPs and nNeurons with 16B06-Gal4. Supplemental Fig. 2. At 24 h T2NB lineages can only be characterized as medial and lateral. (A) 12E09 > UAS-GFP at 24 h targets proliferative T2NBs (GFP+, Pnt + yellow circles). Scale bar 5 μm. (B) Quantification of Syp levels in medial and lateral T2NBs at 24 h. n = 5 brains. Student t-tests were used to compare medial cells to lateral cells. *p < 0.05; **p < 0.01; ***p < 0.001; ****p < 0.0001. Supplemental Fig. 3. Lineage specific Syp levels in T2NBs and nINPs is equivalent except for DL2. (A) Quantification of Syp levels in T2NBs and nINPs in each specific lineage. n = 5 brains. Student t-tests were used to compare medial cells to lateral cells. *p < 0.05; **p < 0.01; ***p < 0.001; ****p < 0.0001. Supplemental Fig. 4. 12E09-Gal4 is expressed in embryonic T2NBs and is required for PF-R and E-PG neuron morphology. (A) 12E09-Gal4 > UAS-GFP in embryonic T2NBs. T2NBs (GFP+ Pnt+, cyan circles). Scale bar 5 μm. (B) Schematic of 12E09-Gal4 expression in embryonic and larval T2NBs and n/yINPs. (C) 12E09-Gal4 > UAS-ImpRNAi turns on earlier in development. T2NBs (cyan circles, GFP- Dpn+), nINPs (yellow circles, GFP- Dpn+), and yINPs (white circles, GFP+ Dpn-) show a loss of Imp at 48 h in T2NBs. Scale bar 5 μm. (D-E) Confocal maximum intensity projections of control, ImpRNAi and ImpOE in PF-R and E-PG neurons. n = 5, scale bar 20 μm. Supplemental Fig. 5. 16B06 > ImpRNAi causes an increase in cell number at 48 h and 72 h. (A-B) Number of oINPs (A) and nNeurons (B) in control, ImpRNAi and ImpOE. Each point is [file 13064_2023_177_MOESM2_ESM.zip › Fig Sup 2.tif]

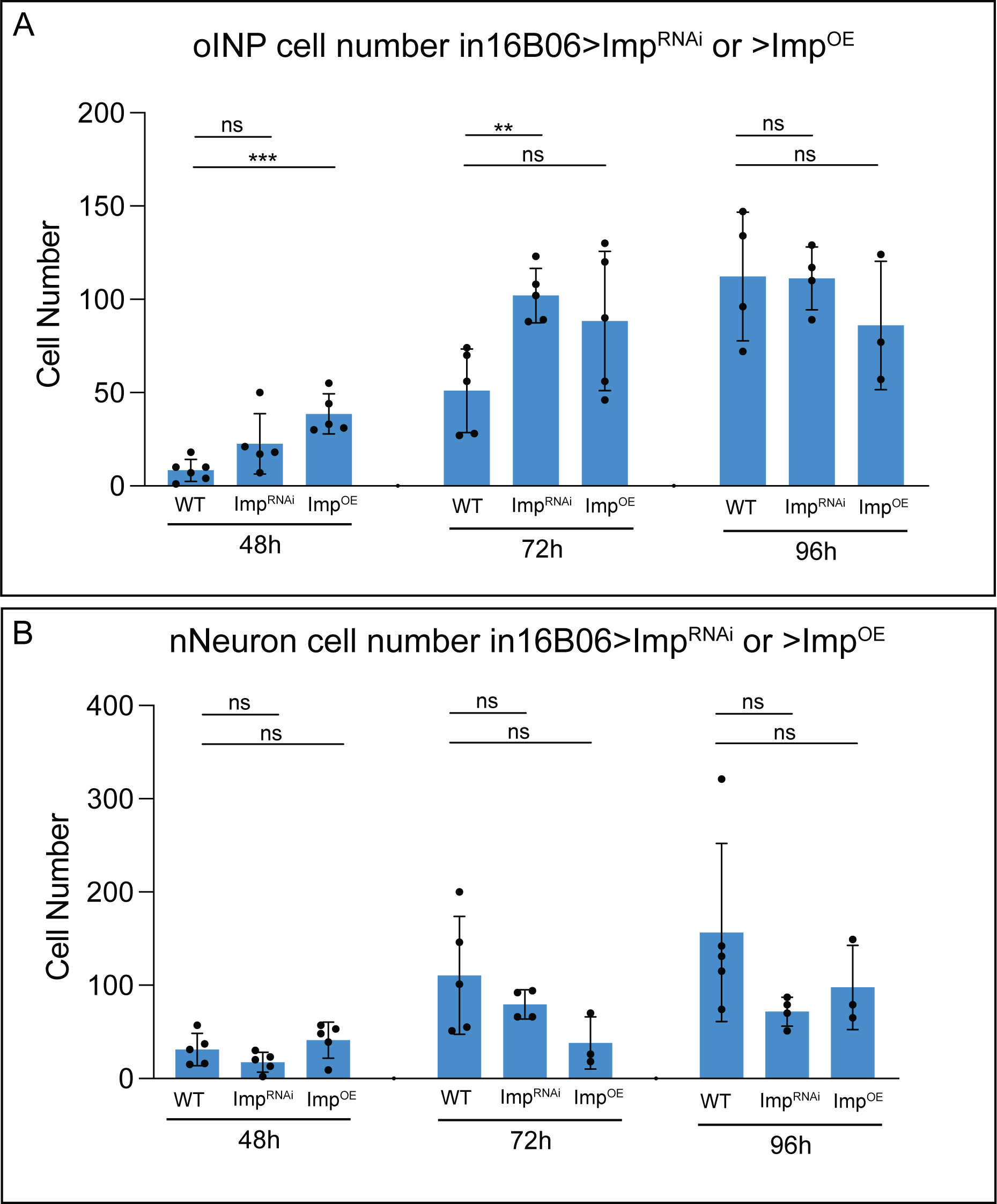

Supplement: Supplementary file 2 — Additional file 2 : Supplemental Fig. 1. INP staging criteria. Schematic showing markers that define different stages in INP lineage progression. T2NBs (green, GFP- Pnt+); nINPs contact the parental NB (purple, GFP- Pnt+); yINPs (yellow, GFP+ Pnt+) border nINPs; mINPs (blue, GFP+ Grh + Scro-); oINPs (pink, GFP+ Grh- Scro+); and nNeurons (orange, GFP+ Elav+ Scro-). GFP was driven in nINPs, yINPs, mINPs and oINPs with 12E09-Gal4, and in oINPs and nNeurons with 16B06-Gal4. Supplemental Fig. 2. At 24 h T2NB lineages can only be characterized as medial and lateral. (A) 12E09 > UAS-GFP at 24 h targets proliferative T2NBs (GFP+, Pnt + yellow circles). Scale bar 5 μm. (B) Quantification of Syp levels in medial and lateral T2NBs at 24 h. n = 5 brains. Student t-tests were used to compare medial cells to lateral cells. *p < 0.05; **p < 0.01; ***p < 0.001; ****p < 0.0001. Supplemental Fig. 3. Lineage specific Syp levels in T2NBs and nINPs is equivalent except for DL2. (A) Quantification of Syp levels in T2NBs and nINPs in each specific lineage. n = 5 brains. Student t-tests were used to compare medial cells to lateral cells. *p < 0.05; **p < 0.01; ***p < 0.001; ****p < 0.0001. Supplemental Fig. 4. 12E09-Gal4 is expressed in embryonic T2NBs and is required for PF-R and E-PG neuron morphology. (A) 12E09-Gal4 > UAS-GFP in embryonic T2NBs. T2NBs (GFP+ Pnt+, cyan circles). Scale bar 5 μm. (B) Schematic of 12E09-Gal4 expression in embryonic and larval T2NBs and n/yINPs. (C) 12E09-Gal4 > UAS-ImpRNAi turns on earlier in development. T2NBs (cyan circles, GFP- Dpn+), nINPs (yellow circles, GFP- Dpn+), and yINPs (white circles, GFP+ Dpn-) show a loss of Imp at 48 h in T2NBs. Scale bar 5 μm. (D-E) Confocal maximum intensity projections of control, ImpRNAi and ImpOE in PF-R and E-PG neurons. n = 5, scale bar 20 μm. Supplemental Fig. 5. 16B06 > ImpRNAi causes an increase in cell number at 48 h and 72 h. (A-B) Number of oINPs (A) and nNeurons (B) in control, ImpRNAi and ImpOE. Each point is [file 13064_2023_177_MOESM2_ESM.zip › Fig Sup 3.tif]

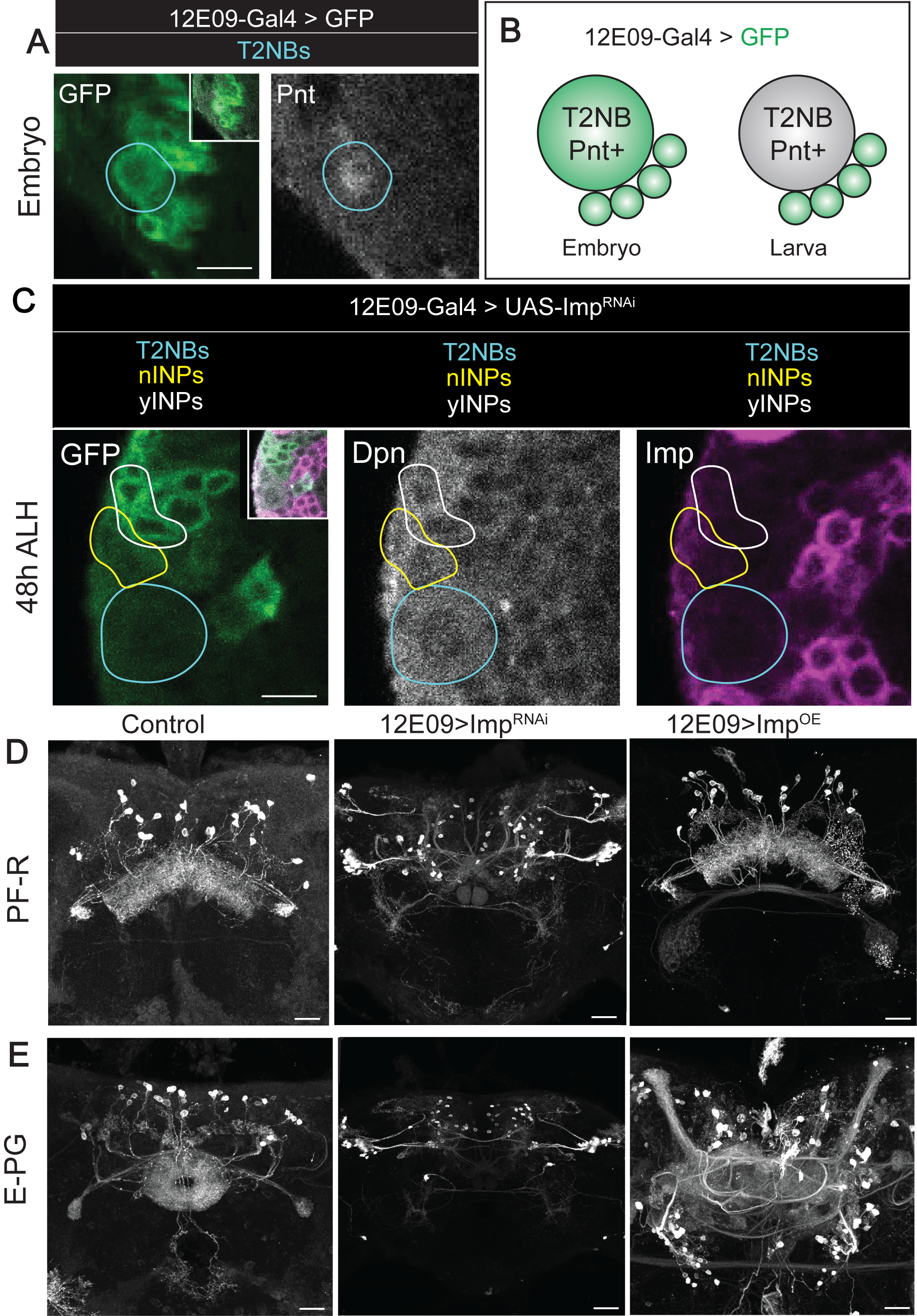

Supplement: Supplementary file 2 — Additional file 2 : Supplemental Fig. 1. INP staging criteria. Schematic showing markers that define different stages in INP lineage progression. T2NBs (green, GFP- Pnt+); nINPs contact the parental NB (purple, GFP- Pnt+); yINPs (yellow, GFP+ Pnt+) border nINPs; mINPs (blue, GFP+ Grh + Scro-); oINPs (pink, GFP+ Grh- Scro+); and nNeurons (orange, GFP+ Elav+ Scro-). GFP was driven in nINPs, yINPs, mINPs and oINPs with 12E09-Gal4, and in oINPs and nNeurons with 16B06-Gal4. Supplemental Fig. 2. At 24 h T2NB lineages can only be characterized as medial and lateral. (A) 12E09 > UAS-GFP at 24 h targets proliferative T2NBs (GFP+, Pnt + yellow circles). Scale bar 5 μm. (B) Quantification of Syp levels in medial and lateral T2NBs at 24 h. n = 5 brains. Student t-tests were used to compare medial cells to lateral cells. *p < 0.05; **p < 0.01; ***p < 0.001; ****p < 0.0001. Supplemental Fig. 3. Lineage specific Syp levels in T2NBs and nINPs is equivalent except for DL2. (A) Quantification of Syp levels in T2NBs and nINPs in each specific lineage. n = 5 brains. Student t-tests were used to compare medial cells to lateral cells. *p < 0.05; **p < 0.01; ***p < 0.001; ****p < 0.0001. Supplemental Fig. 4. 12E09-Gal4 is expressed in embryonic T2NBs and is required for PF-R and E-PG neuron morphology. (A) 12E09-Gal4 > UAS-GFP in embryonic T2NBs. T2NBs (GFP+ Pnt+, cyan circles). Scale bar 5 μm. (B) Schematic of 12E09-Gal4 expression in embryonic and larval T2NBs and n/yINPs. (C) 12E09-Gal4 > UAS-ImpRNAi turns on earlier in development. T2NBs (cyan circles, GFP- Dpn+), nINPs (yellow circles, GFP- Dpn+), and yINPs (white circles, GFP+ Dpn-) show a loss of Imp at 48 h in T2NBs. Scale bar 5 μm. (D-E) Confocal maximum intensity projections of control, ImpRNAi and ImpOE in PF-R and E-PG neurons. n = 5, scale bar 20 μm. Supplemental Fig. 5. 16B06 > ImpRNAi causes an increase in cell number at 48 h and 72 h. (A-B) Number of oINPs (A) and nNeurons (B) in control, ImpRNAi and ImpOE. Each point is [file 13064_2023_177_MOESM2_ESM.zip › fig sup 4.tif]

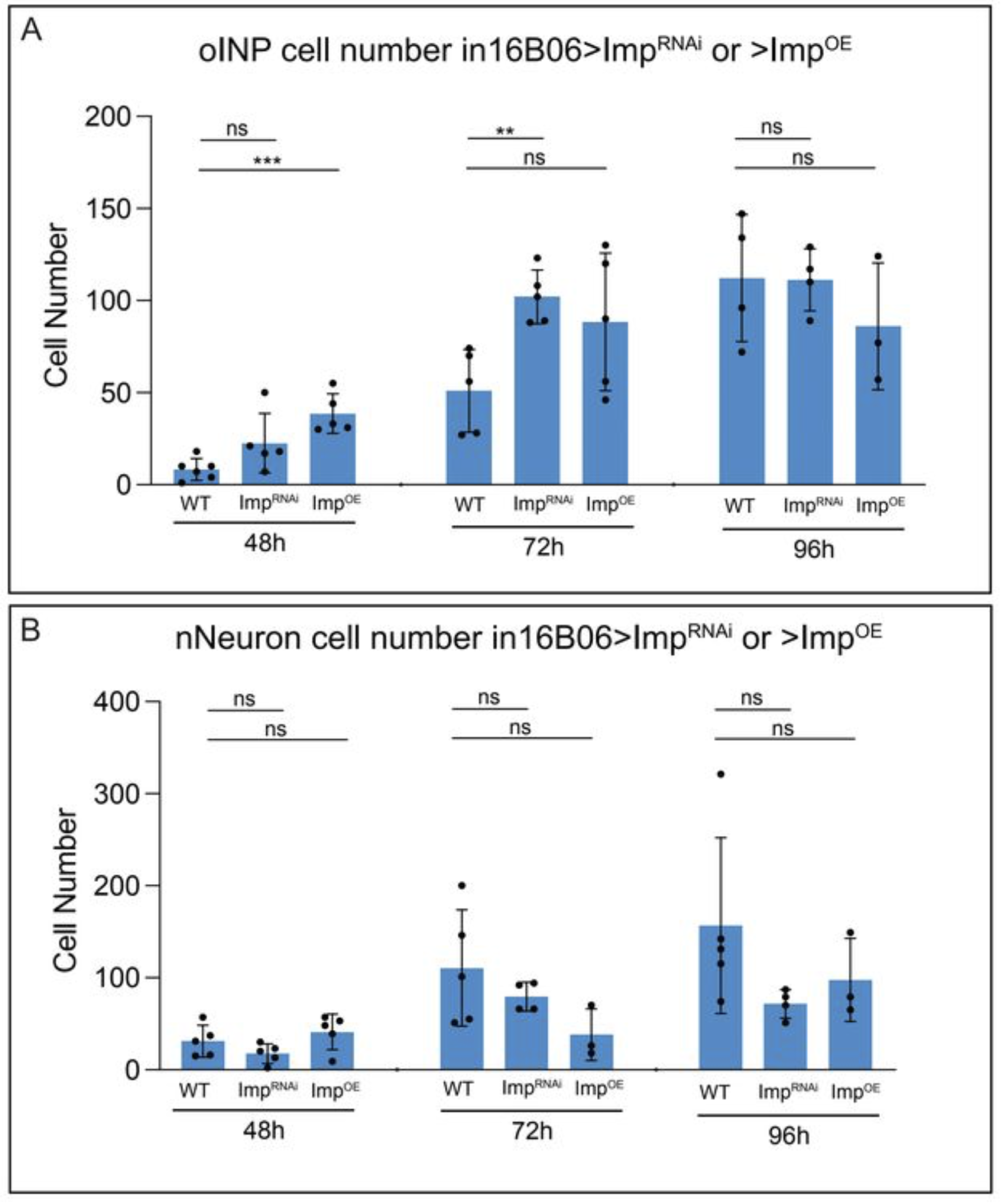

Supplement: Supplementary file 2 — Additional file 2 : Supplemental Fig. 1. INP staging criteria. Schematic showing markers that define different stages in INP lineage progression. T2NBs (green, GFP- Pnt+); nINPs contact the parental NB (purple, GFP- Pnt+); yINPs (yellow, GFP+ Pnt+) border nINPs; mINPs (blue, GFP+ Grh + Scro-); oINPs (pink, GFP+ Grh- Scro+); and nNeurons (orange, GFP+ Elav+ Scro-). GFP was driven in nINPs, yINPs, mINPs and oINPs with 12E09-Gal4, and in oINPs and nNeurons with 16B06-Gal4. Supplemental Fig. 2. At 24 h T2NB lineages can only be characterized as medial and lateral. (A) 12E09 > UAS-GFP at 24 h targets proliferative T2NBs (GFP+, Pnt + yellow circles). Scale bar 5 μm. (B) Quantification of Syp levels in medial and lateral T2NBs at 24 h. n = 5 brains. Student t-tests were used to compare medial cells to lateral cells. *p < 0.05; **p < 0.01; ***p < 0.001; ****p < 0.0001. Supplemental Fig. 3. Lineage specific Syp levels in T2NBs and nINPs is equivalent except for DL2. (A) Quantification of Syp levels in T2NBs and nINPs in each specific lineage. n = 5 brains. Student t-tests were used to compare medial cells to lateral cells. *p < 0.05; **p < 0.01; ***p < 0.001; ****p < 0.0001. Supplemental Fig. 4. 12E09-Gal4 is expressed in embryonic T2NBs and is required for PF-R and E-PG neuron morphology. (A) 12E09-Gal4 > UAS-GFP in embryonic T2NBs. T2NBs (GFP+ Pnt+, cyan circles). Scale bar 5 μm. (B) Schematic of 12E09-Gal4 expression in embryonic and larval T2NBs and n/yINPs. (C) 12E09-Gal4 > UAS-ImpRNAi turns on earlier in development. T2NBs (cyan circles, GFP- Dpn+), nINPs (yellow circles, GFP- Dpn+), and yINPs (white circles, GFP+ Dpn-) show a loss of Imp at 48 h in T2NBs. Scale bar 5 μm. (D-E) Confocal maximum intensity projections of control, ImpRNAi and ImpOE in PF-R and E-PG neurons. n = 5, scale bar 20 μm. Supplemental Fig. 5. 16B06 > ImpRNAi causes an increase in cell number at 48 h and 72 h. (A-B) Number of oINPs (A) and nNeurons (B) in control, ImpRNAi and ImpOE. Each point is [file 13064_2023_177_MOESM2_ESM.zip › Fig Sup 5.tif]
